# A Novel Narrative E-Writing Intervention for Parents of Children With Chronic Life-Threatening Illnesses: Protocol for a Pilot, Open-Label Randomized Controlled Trial

**DOI:** 10.2196/17561

**Published:** 2020-07-05

**Authors:** Andy Hau Yan Ho, Oindrila Dutta, Geraldine Tan-Ho, Toh Hsiang Benny Tan, Xinyi Casuarine Low, Sashikumar Ganapathy, Josip Car, Ringo Moon-Ho Ho, Chun Yan Miao

**Affiliations:** 1 Psychology Program School of Social Sciences Nanyang Technological University Singapore Singapore; 2 Lee Kong Chian School of Medicine Nanyang Technological University Singapore Singapore; 3 Palliative Care Centre for Excellence in Research and Education Singapore Singapore; 4 School of Computer Science and Engineering Nanyang Technological University Singapore Singapore; 5 Club Rainbow Singapore Singapore; 6 Centre for Population Health Sciences Lee Kong Chian School of Medicine Nanyang Technological University Singapore Singapore

**Keywords:** narrative therapy, psychotherapy, pediatric palliative care, end-of-life care, randomized controlled trial, cyber-counseling, mobile phone

## Abstract

**Background:**

A novel evidence-based Narrative e-Writing Intervention (NeW-I) has been developed and tested in Singapore to advance psychosociospiritual support for parents of children with chronic life-threatening illnesses. NeW-I is informed by an international systematic review and a Singapore-based qualitative inquiry on the lived experience of parental bereavement and supported by literature on anticipatory grief interventions for improving the holistic well-being of parent caregivers of seriously ill children.

**Objective:**

This study's aim was to provide an accessible platform, NeW-I—which is a strengths- and meaning-focused and therapist-facilitated mobile app and web-based counseling platform—that aims to enhance quality of life, spiritual well-being, hope, and perceived social support and reduce depressive symptoms, caregiver burden, and risk of complicated grief among parents of children with chronic life-threatening illnesses.

**Methods:**

The NeW-I therapist-facilitated web-based platform comprises a mobile app and a website (both of which have the same content and functionality). NeW-I has been implemented in Singapore as a pilot open-label randomized controlled trial comprising intervention and control groups. Both primary and secondary outcomes will be self-reported by participants through questionnaires. In collaboration with leading pediatric palliative care providers in Singapore, the trial aims to enroll 36 participants in each group (N=72), so that when allowing for 30% attrition at follow-up, the sample size will be adequate to detect a small effect size of 0.2 in the primary outcome measure, with 90% power and two-sided significance level of at least .05. The potential effectiveness of NeW-I and the accessibility and feasibility of implementing and delivering the intervention will be assessed.

**Results:**

Funding support and institutional review board approval for this study have been secured. Data collection started in January 2019 and is ongoing.

**Conclusions:**

NeW-I aspires to enhance holistic pediatric palliative care services through a structured web-based counseling platform that is sensitive to the unique cultural needs of Asian family caregivers who are uncomfortable with expressing emotion even during times of loss and separation. The findings of this pilot study will inform the development of a full-scale NeW-I protocol and further research to evaluate the efficacy of NeW-I in Singapore and in other Asian communities around the world.

**Trial Registration:**

ClinicalTrials.gov NCT03684382; https://clinicaltrials.gov/ct2/show/NCT03684382

**International Registered Report Identifier (IRRID):**

DERR1-10.2196/17561

## Introduction

### Background

There is a global increase in the number of children living with chronic life-threatening illnesses [1,2]. In Singapore, child deaths (age <19 years) due to chronic conditions increased from 204 per year to 245 per year during the 2014 to 2016 period [3]. In fact, congenital anomalies as well as cardiovascular and cerebrovascular diseases account for over half of all deaths in the 0 to 19 years age range in Singapore [4]. Due to technological developments in medical care, children with chronic life-threatening illnesses can live longer, but they simultaneously face the challenge of being dependent on their caregivers (usually, their parents) and disability for a prolonged period [1]. However, not all seriously ill children and their parents receive adequate palliative support services [3].

Being a parent caregiver of a child with a chronic life-threatening illness is a highly stressful experience [5]. In addition to the typical challenges of parenting, parent caregivers must navigate a complex web of stressors, including the practical and financial burden of caregiving [6], strained marital and social relationships [7], and neglect of other healthy children and family members [8]. Furthermore, parent caregivers of children with chronic life-threatening illnesses need to frequently communicate and engage with medical professionals, but such interactions can increase the anxiety and distress of the parents if they are not adequately involved in making decisions regarding their child’s treatment [9,10]. Thus, the stressors associated with caregiving and the elevated demand on resources put parent caregivers at increased risk of depressive symptoms, fatigue, and overall poor quality of life [11].

### Parental Bereavement Trajectories of Child Loss

**Figure 1:**
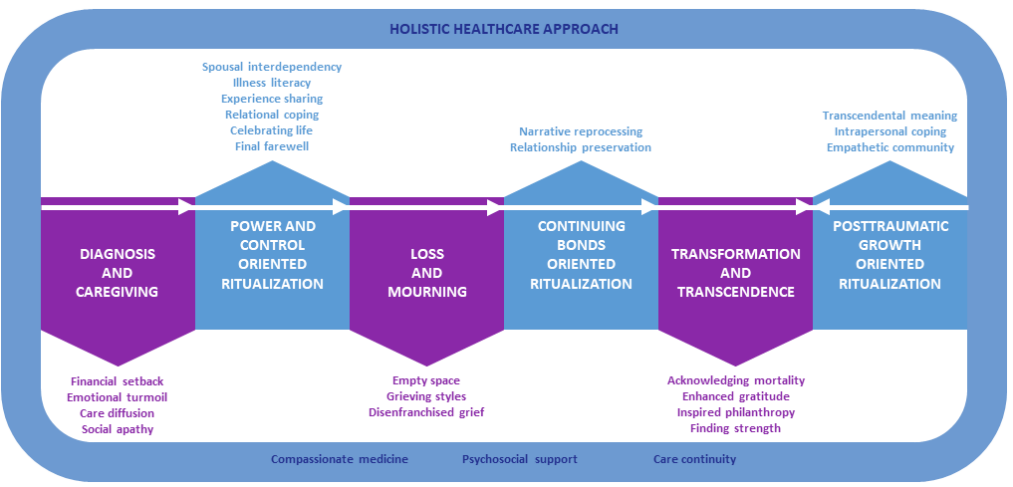
Trauma to transformation model of parental bereavement in Asia.

Our research team recently conducted a qualitative systematic review of 25 high-quality research papers published between 2000 and 2017, exploring the lived experience of parental bereavement due to a child’s chronic life-threatening illness. A 4-phase parental bereavement trajectory of child loss was developed, highlighting appropriate interventions that help parents identify care needs, elicit caregiving strengths, enhance death preparedness, and foster meaning-making throughout the trajectory of their child’s illness to reduce psychoemotional distress during the period of end-of-life caregiving and into bereavement [12]. Our research team conducted a second study to examine the Asian experience of parental bereavement via meaning-oriented strength-focused interviews with 6 couples, 13 mothers, 4 fathers, and 2 primary parental figures (N=25 parental units) who lost their child to a chronic life-threatening illness in Singapore [13,14]. Grounded theory analysis revealed 7 themes and 25 subthemes that were organized into a trauma-to-transformation model of parental bereavement (Figure 1). This culture-specific model shows the milestones of how Asian bereaved parents journeyed through their child’s life-threatening illness and eventual death, describing the ritualistic actions as well as family communication and transactional patterns that aided them to better cope with their loss, to regain control over their lives, to sustain a continuing bond with their deceased child, and to move forward with and ultimately transcend their grief through meaning reconstruction [13,14].

These findings echo previous literature [15-18] positing that parents facing potential child loss could benefit from psychosocial and therapeutic interventions as soon as prognosis is known and throughout the trajectory of the illness, which could ease the transition from caregiving through mortality and bereavement, thus mitigating adverse grief outcomes (Table 1). However, most supportive interventions for parents caring for children with chronic life-threatening illnesses only occur after bereavement [19-21], and a recent systematic review found negligible evidence to support their effectiveness [22]. As such, there is a need to develop a preloss intervention to augment pediatric palliative care and parental bereavement support service—one that empowers parents to reflect on their caregiving experiences, explore and identify resources that could help them better cope with the challenges of caregiving, and support their child to live a meaningful life despite a chronic life-threatening illness.

**Table 1:** Clinical implications of findings from the qualitative study on the lived experience of parental bereavement in Asia.

Findings from qualitative studies on the lived experience of parental bereavement in Asia	Implications for clinical work
Facing their child’s impending mortality is a difficult experience, which can isolate parents. Societal attitudes toward illness can make it difficult for parents to engage with their previous social networks.	Support parents in gaining a greater sense of control over their lives and strengthening resilience during the period of end-of-life caregiving through empathic support and psychoeducational resources for self-care and healthy coping.
Parents seek to understand the medical terminology associated with their child’s illness and prognosis of the condition, so that they can evaluate potential risks and benefits of treatment procedures and make informed care decisions.	Empower parents to provide the best possible care to their child through exploration of resources for seeking information about illness and caregiving.
Parents desire to give their children a chance to rise above the difficulties brought on by the illness and display strength to help their child live as fully as possible.	Facilitate meaningful family experiences that allow parents and their children to move away from the drudgery of illness, suffering, and caregiving and focus on building parent-child memories.
Asian parents tend to have a collaborative approach to caregiving for their sick child. They often rely on family members, relatives, and other parents of sick children for support.	Explore sources of support, which participants have within their close social network and how they can be harnessed in care provision.

### Elements of a Preloss Intervention for Parents of Children With Chronic Life-Threatening Illnesses

In developing a preloss intervention that could meaningfully impact families throughout their child’s trajectory of illness and leading to the final days of their child’s life, a number of important therapeutic elements need to be considered and incorporated. First, anticipatory grief, defined as the process of mourning the loss of a loved one before actual loss that enables caregivers to experience and adjust to various grief responses, must be central to such an intervention [17,23]. Anticipatory grief can smoothen the process of coping with death as the individual has the scope to come to terms with the loss in advance [24]. Studies have found that strength-based end-of-life interventions with elements that address anticipatory grief can improve adult patients’ quality of life and mitigate poor bereavement outcomes among family caregivers [25]. It is, therefore, possible that an anticipatory grief-based psychotherapeutic intervention for parents of children with chronic life-threatening illnesses could help parents to understand and regulate their emotions, enhance death preparedness, and thereby build resilience.

Second, it would be useful for a preloss intervention for parents of children with chronic life-threatening illnesses to adopt a meaning reconstruction approach [26-28], with each individual actively constructing a phenomenological world of their experiences in relation to various familial and sociocultural contexts and supporting their sense of loss and grief accordingly. Such a meaning reconstruction approach empowers grievers to choose whether to direct their attention to the loss and process turbulent feelings or to focus on practical adjustments to re-engage with their everyday life. Third, a preloss intervention would benefit from a narrative approach [29,30], which could help individuals connect with emotions that are challenging to accept, generate new meaningful stories about life and loss, and restructure negative emotional appraisal of situations such as end-of-life caregiving into more positive ones, thereby generating a sense of hope.

Two effective examples of applying the meaning reconstruction approach and the narrative approach in supporting holistic end-of-life care are dignity therapy [25,31,32] and family dignity intervention [33]. Both of these are evidence-based psychotherapies that address the physical, psychosocial, and existential issues pertaining to one’s dignity at the end-of-life period. Specifically, a family dignity intervention is designed to support the collective experience of grief and loss for Asian families facing mortality, and it could add great value to a preloss intervention for parents of children with chronic life-threatening illnesses. In practice, family dignity intervention comprises a meaning-focused interview with a patient-and-family caregiver dyad that fosters the expression of appreciation and emotional connection through the retelling of important life narratives. The interview is recorded, transcribed, and edited into a legacy document and returned to the dyad for sharing with their loved ones for healing and remembrance.

Finally, a preloss intervention for parents of children with chronic life-threatening illnesses must be mindful of the caregiving responsibilities and limitations that serve as barriers for parents to engage in sit-and-talk therapy [12]. It is possible that a web-based platform that is cost-effective and time-efficient [34] can deliver psychotherapy to such parents. Such therapist-facilitated internet-based platforms are increasingly used for brief and effective psychotherapy for a range of conditions, including depression, anxiety, and stress [35-40]. Moreover, when web-based platforms use writing as the modality for emotional expression and reflection, efficacy is superior compared with audio or video mediums [41]. Finally, the anonymity of an e-writing channel can encourage greater willingness to self-disclose [42,43].

### This Study and Objectives

Globally, pediatric palliative care interventions predominantly emphasize the stages of grief and psychological tasks that grieving parents must accomplish after their child’s death [22], and in Singapore, there is no known empirically tested intervention to provide psychoemotional support and psychoeducational resources to parents of children with chronic life-threatening illnesses. Singapore is a leading nation in digital readiness, smartphone utilization for communication is ingrained into the everyday life of its people [44], and web-based intervention services for improving well-being have been welcomed by the Singaporean community [45,46]. Hence, it is reasonable to propose that web-based solutions are vitally useful for enhancing pediatric palliative care and parental bereavement support services. Furthermore, in view of the fact that Asian family caregivers can be uncomfortable with explicit emotional expression even during times of loss and separation [47], a web-based platform and the relative anonymity it offers to participants [43] would be appropriate for an Asian population.

The research team of this study integrated the aforementioned elements necessary for a preloss intervention for parents of children with chronic life-threatening illnesses and conceived a Narrative e-Writing Intervention (NeW-I) to address the gap in pediatric palliative care delivery and research in the local context. NeW-I is a novel web-based, therapist-facilitated, strength-focused, and meaning-oriented intervention designed to provide direct service to parents of children with chronic life-threatening illnesses. The development and evaluation of NeW-I is guided by the Medical Research Council Framework for the Development and Evaluation of Complex Interventions, which is widely recognized in the design and evaluation of complex interventions to improve health outcomes [48,49]. NeW-I is also inspired by the meaning reconstruction model [50], the narrative approach to anticipatory grief [51], dignity therapy [31,32], family dignity intervention for holistic end-of-life care [33], and the findings of a recent investigation on Asian parental bereavement experience of child loss by our research team [13,14].

The overarching goal of the novel NeW-I intervention model is to provide an accessible platform for parent caregivers to reflect on their experiences of caring for a child with a chronic life-threatening illness from a new perspective (ie, new eye) as well as to create a renewed sense of self-understanding and empowered identity in the context of their experience (ie, new I). The specific research objectives are three-fold: (1) to develop a standardized protocol for a cultural-specific and meaning-oriented NeW-I for anticipatory grief and bereavement support for parent caregivers of children with chronic life-threatening illnesses and eventual death; (2) to evaluate the efficacy of NeW-I in enhancing quality of life, spiritual well-being, hope, and social support as well as decreasing caregiver burden, depressive symptoms, and risk of complicated grief among parent caregivers; and (3) to examine the challenges and pitfalls in the design and implementation of NeW-I through an integrated feasibility and acceptability study for informing large-scale implementation of the intervention.

## Methods

### Study Design and Hypotheses

This study utilizes an open-label randomized controlled trial design comprising 2 groups: (1) an intervention group (structured NeW-I protocol) and (2) a control group (journaling activity unrelated to their child’s illness). It is hypothesized that intervention participants who successfully complete NeW-I will experience an enhanced quality of life, spiritual well-being, sense of hope, and perceived social support and decreased depressive symptoms, subjective caregiver burden, and risk of complicated grief as compared with control participants. It is also hypothesized that NeW-I is deemed as an accessible and user-friendly service by participants.

### Sample

The sample comprises 72 individual parents of varying ethnicity in Singapore (N=72). Where both parents of a child participate in the study, their group allocation is randomly determined, and assessments are completed by both parents independently. To be eligible for participation in this study, the individual must be a parent whose child has been diagnosed with a chronic life-threatening illness and has a prognosis of more than 3 months at the time of enrollment. For the purpose of this study, a *child* has been defined as children and young persons between the ages of 0 and 19 years [52]. The individual must be able to speak, read, and write in English and provide informed consent. Individuals are excluded from this study if they are suffering from severe depressive symptoms and psychological distress, as identified by 2 screening tools. Specifically, to protect participants’ well-being during the pilot testing of NeW-I, those who do not meet the stated cutoff scores of the Patient Health Questionnaire – 9 (PHQ-9; >19) and Kessler psychological distress scale (K-10; >29) are excluded, as formal treatment and therapy would be more beneficial [53,54]. Additionally, if participants cease to meet the inclusion criteria during the study (eg, due to their child’s untimely death), they are excluded from the study and provided with alternative resources for psychosocial support. However, the data that have been collected until the time of their participation will be kept and analyzed so that a complete and comprehensive evaluation of the study will be possible.

### Sample Size Calculation

For a main trial designed with 90% power and two-sided significance level of at least .05, a pilot sample size of 25 per arm is needed to detect a small effect size of 0.2 in the primary outcome measure [55]. A meta-analysis suggested that many high-quality psychotherapy studies (high quality studies defined by a proficiently trained therapist, treatment integrity, N>50) on the treatment of depression have a mean effect size of 0.22 [56]. Allowing for an attrition rate of 30% at follow-up (a larger estimate due to end-of-life context), the sample size must be inflated by a factor of 1/(1−0.3)=1.43. Therefore, the minimum sample size required for this study was 72 or 36 in each group.

### Recruitment Procedures

Purposive sampling was adopted to achieve a target sample size of 72. Potential participants are identified and contacted by the collaborating organization (leading pediatric palliative care providers in Singapore, including KK Women’s and Children’s Hospital, Club Rainbow Singapore, Muscular Dystrophy Association Singapore, and Rare Disorders Society Singapore) to introduce the study to their beneficiaries. This sampling strategy allows the recruitment of participants whose children suffer from a wide range of chronic life-threatening conditions, thus allowing for maximum variation in the sampling. If verbal consent is obtained from potential participants, their contact details are passed to the research team at Nanyang Technological University, who subsequently establish telephone contact, explain study procedures, and introduce the NeW-I web-based platform. All personal information pertaining to potential participants are kept confidential, and only the responsible researchers have access to such information.

Open recruitment is also carried out so that all parents of children with chronic life-threatening illnesses have equal opportunity to participate in a potentially beneficial study and to examine the feasibility of implementing this free and easily accessible intervention in the community. Posters have been placed in strategic locations across Singapore (such as offices of leading pediatric palliative care providers) that provide information regarding the study. When interested participants contact the research team, the study procedures are explained, the NeW-I platform is introduced, and registration information is provided.

### App and Intervention Procedure

The NeW-I therapist-facilitated web-based platform comprises a mobile app and a website (both of which have the same content and functionality). Participants can download the app free of cost from the Apple App Store and the Google Play Store by keying in the relevant keywords or scanning the quick response code provided on the NeW-I study advertisement posters. The NeW-I website is under construction. As shown in Multimedia Appendix 1, when participants initially log in to the app, they are directed to a study participation and informed consent page that provides details about study procedures, institutional affiliations of the research team, rights of research participants, and protection of confidentiality. After participants endorse this web-based informed consent form on the NeW-I platform, they are directed to a demographic information page. Participants are also requested to provide their contact details and last few digits of their national identification number to ensure that only 1 user account is created by each individual.

**Figure 2:**
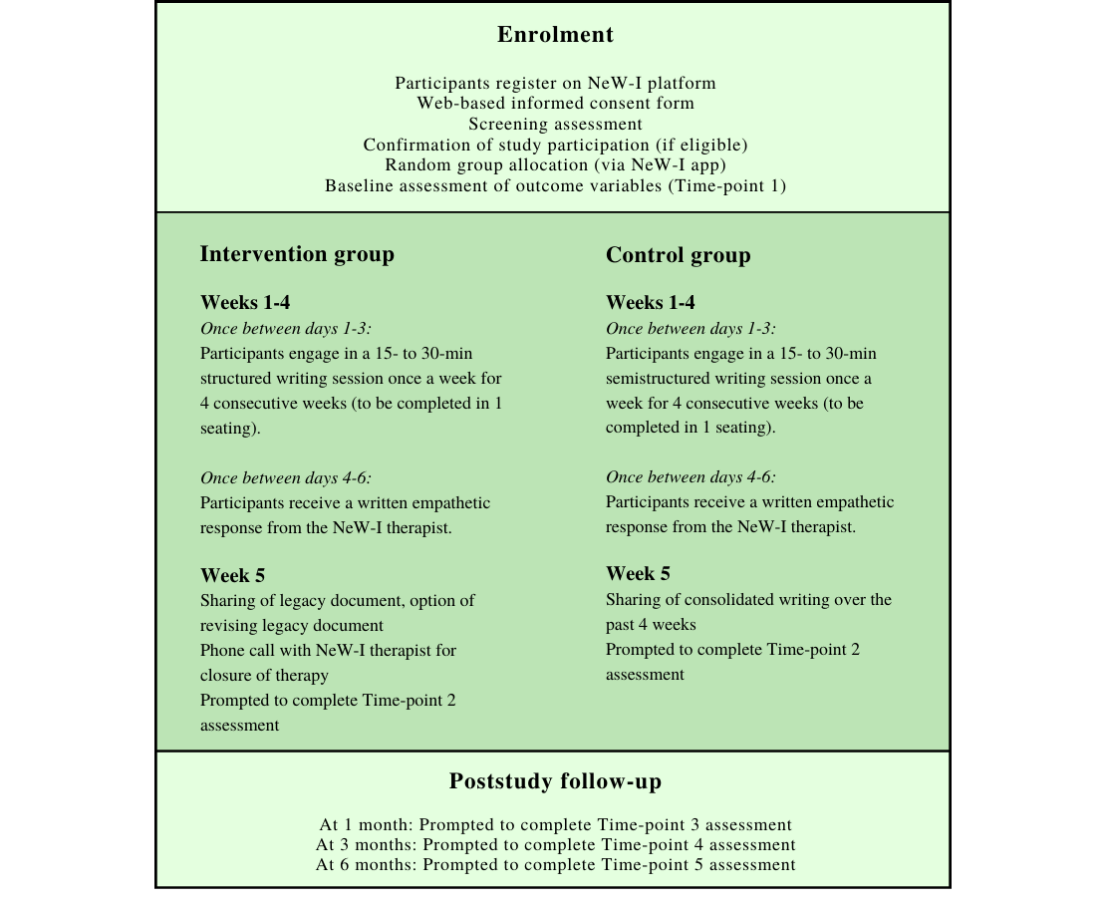
Description of Narrative e-Writing Intervention (NeW-I) procedures.

Following this, participants are directed to a screening page where participants complete the PHQ-9 and the K-10. Those who pass the screening assessments receive confirmation of study participation and are requested to wait for a phone call from the research team. Those who do not pass the screening assessments are thanked for their time and provided with resources for psychosocial support. Individuals who pass the screening assessments receive a phone call from the NeW-I team as a means of identity checking. Following this, participants complete baseline measures (time point 1 assessment). This is followed by a random allocation of participants to either the intervention group or the control group, which is performed via the NeW-I platform using computer-generated random numbers. Participants are then directed to the first writing session. Participants have the option to choose to begin the first writing session immediately or delay for a maximum of 3 days. The day on which participants begin their first writing session is day 1 of week 1. Participants who do not begin their first writing session within 3 days are excluded from the study. However, a concession period of 7 days is given to participants who have a genuine reason for their inability to adhere to the protocol (such as unexpected changes in their child’s health condition) and have expressed keen interest in participating in the study. A similar concession period of 7 days is also maintained for participants who are unable to adhere to the study protocol in weeks 2, 3, and 4 (such that each participant can only be given 1 concession during the entire duration of their study participation). Detailed study procedures are described in Figure 2.

NeW-I is delivered by trained therapists in the research team who are experts in death education and grief counseling and have the clinical competence to work with family caregivers in pediatric palliative settings. All team members have successfully completed research integrity modules under the provisions of Nanyang Technological University’s institutional review board and adhere to the board’s guidelines for safeguarding participants’ identity and confidentiality.

### Intervention and Control Groups

Both intervention and control group participants follow the procedures described in Figure 2. There are 4 weekly sessions of writing. A template has been provided to ensure that the participants’ writings tie in with the session objectives. To improve participants’ adherence to the study protocol, they receive an automated notification on their phone app and email each time a fresh writing session becomes available to them. Participants were assured of anonymity and confidentiality of their writing to encourage open and honest self-expression. The structured writing for each session requires 15 to 30 min, as exposure and time to process ideas through written disclosure over at least three sessions of 15 min each can produce effective outcomes [57].

For the intervention group participants, each weekly session has a unique objective (Table 2). Briefly, in week 1, participants reflect on the demands of caring for a child with a chronic life-threatening illness and the means to cope with these challenges. In week 2, participants consider avenues where they could seek more information about their child’s illness and resources for caregiving. In week 3, participants examine the sources of support that they have within their network of family and friends. In week 4, participants explore how they (and their children) could rise above illness-related challenges and live their lives as fully as possible.

For control group participants, the objective is consistent across the 4 weeks, that is, participants engage in a weekly writing session that is unrelated to their child’s illness and allows them to experience the therapeutic benefits of narrative writing (Table 3).

**Table 2:** Content and questions for reflective writing for intervention group.

Session structure	Week 1	Week 2	Week 3	Week 4
Objective	To provide participants with a platform to reflect on the emotional, practical, and financial demands of caring for a child with a chronic life-threatening illness and the means to cope with these challenges.	To explore avenues where participants can seek more information about their child’s illness and resources for caregiving.	To explore the sources of support that participants have within their close network of family and friends.	To explore how participants (and their children) can rise above illness-related challenges and live their lives as fully as possible.
Questions for reflective writing	Tell us a little about your child and what you love about him or her. Tell us about the challenges that you have encountered when caring for your child. What has been your biggest challenge so far? How have you coped with it? What are three things that have helped you cope in your caregiving journey?	How satisfied are you with the knowledge and information that you have about your child’s condition? What has been helpful in providing you with the knowledge and information? What are some forms of support that have been helpful for you in providing quality care to your child? How were these forms of support helpful? What would help you to feel more competent as your child’s caregiver? What is one thing you could do to make that difference?	Tell us about some people who have been helpful or supportive in your caregiving journey. How have they helped you to cope during difficult times? On a scale of 1 to 10, with 1 being “Not at All” and 10 being “Very Much,” how satisfied are you with your spousal relationship? (Please omit this question if it does not apply to you.) What might make that score a little higher? What are some things that others could do for you that could further help you in your caregiving journey?	Tell us what you love best about your child. What quality about him or her makes you proud? What would a good day for your child look like right now? What makes it a good day? Tell us about an enjoyable moment with your child. What are some things you can do to make an enjoyable moment happen?
Counseling goals	To affirm the strengths that have helped participants to survive and thrive. To provide psychoeducation about local social welfare organizations that can provide them with support.	To acknowledge participants’ efforts to seek power and control over their seemingly uncontrollable lives through illness literacy. To provide psychoeducation about sources for seeking more information about their child’s illness, treatment options, and resources for caregiving.	To reframe that they are indeed blessed to have the support of their spouse or family or friends to help them to cope with this challenging period. To reframe participants’ sharing from sessions 1, 2, and 3 by taking the semantic content as it is but providing an alternative viewpoint of perceiving the situation.	To assist participants (and their children) in building meaningful and cherished memories through reflecting on achievements and fulfillment of dreams. To examine ways in which participants can enhance quality of life in their child’s final days.

**Table 3:** Content and questions for reflective writing for control group.

Session structure	Week 1	Week 2	Week 3	Week 4
Questions for reflective writing	This week, we’d love to know a little bit about you. Tell us what an average day in your life looks like. Feel free to share with us any and every detail that you find comfortable to talk about!	This week, we’d like to know about what has the past week been like for you? Feel free to share with us in as much detail as you like!	This week, we’d like to know what is the biggest challenge (e.g. emotional, financial, practical etc.) that you are facing right now? Do feel free to add on anything else about this challenge that you would like us to know!	This week, we’d like to know what you find most comforting right now (e.g. family, relationships, work, hobbies and other activities etc.). Tell us about what makes this thing comforting for you?

### Evaluation of Outcomes

#### Quantitative Assessments

Via the NeW-I platform, both intervention and control group participants fill out a sociodemographic form at baseline and are then assessed on a battery of self-reported standardized and validated measures across 5 time points. The primary outcome measure is the participants’ quality of life, as measured by the Kemp Quality of Life scale (KQOL) [58]. Secondary outcomes are assessed using (1) a modified version of the Functional Assessment of Chronic Illness Therapy - Spiritual Well-Being scale (FACIT-Sp-12) [59], (2) the Herth Hope Index (HHI) [60], (3) Patient Health Questionnaire – 9 [53], (4) the Burden Scale for Family Caregivers-Short (BSFC-s) [61], (5) the Inventory of Social Support (ISS) [62], and (6) a modified version of the Brief Grief Questionnaire (BGQ) [63] (Table 4).

**Table 4:** Quantitative outcome measures.

Measure	Number of items	Rating scale; factor
Kemp Quality of Life scale	1	7-point Likert scale; 1 factor
Functional Assessment of Chronic Illness Therapy - Spiritual Well-Being (adapted version)	12	5-point Likert scale; 3 factors: meaning, peace, and faith
Herth hope index	12	4-point Likert scale; 1 factor
Patient Health Questionnaire - 9 (also serves as a screening assessment)	9	4-point Likert scale; 1 factor
Burden Scale for Family Caregivers-Short	10	4-point Likert scale; 1 factor
Inventory of Social Support	5	5-point Likert scale; 1 factor
Brief Grief Questionnaire (adapted version)	5	3-point Likert scale; 1 factor

Assessment for both intervention and control groups takes place at 5 time points: baseline (time point 1), immediately after completion of the intervention or control protocol (time point 2), 1 month after completion of the intervention or control protocol (time point 3), 3 months after completion of the intervention or control protocol (time point 4), and a final follow-up 6 months after completion of the intervention or control protocol (time point 5). Participants receive an automated notification on their phone app and email each time an assessment set becomes available to them. For each completed assessment, participants receive a voucher worth Singapore $30 (approximately US $21).

#### Acceptability and Feasibility Study

To evaluate the acceptability and effectiveness of NeW-I, all intervention participants are invited to participate in a semistructured interview at the completion of all intervention components at time point 2, which explores the impact of the intervention, aspects of the intervention found to be helpful, aspects of the intervention found to be unhelpful and how they could be improved, challenges encountered in completing the intervention, and scope for enhancing intervention usability. To assess the feasibility of implementing and delivering NeW-I, the research team maintains an audit trail of the time needed to provide feedback to participants and restructure their narrative writing; deviations from the intervention protocol (if any); uncompleted interventions and their reasons; and NeW-I therapists’ perceptions of competence, observations of participants’ experiences and responses, and difficult or deviant cases. All feedback provided to participants will be vetted by at least two members of the research team for data monitoring, quality, and safety assurance.

### Data Analysis

#### Quantitative Data

The statistical analysis plan for the quantitative data in this study was designed according to the Consolidated Standards of Reporting Trials guidelines [64] and recommendations made by Gamble et al [65] for clinical trials. Quantitative parameters will be presented as mean (SD) or median (IQR), and categorical variables will be presented as numbers and proportions. The internal consistency of psychometric scales will be assessed using Cronbach alpha. To compare the primary quantitative outcome (KQOL) and secondary quantitative outcomes (FACIT-Sp-12, HHI, PHQ-9, BSFC-s, ISS, and BGQ) between the intervention and control groups over time (time point 1 to time point 5, with time point 2 being the primary time point of comparison), multilevel mixed-effect regression analysis will be used with time as a random effect. All demographic and other relevant covariates measured at baseline will be adjusted in the model. Mixed models allow us to include all available data in the presence of dropouts occurring into the analysis [66]. Individual multivariable models will be constructed for each outcome to estimate the effect sizes and corresponding CIs. Clinically meaningful interactions will also be checked and included in the model if *P*<.10. Marginal effects will be used to predict mean outcome scores for the intervention and control groups. For this study, a two-sided significance level of at least .05 will be considered a statistically significant finding. All analyses will be conducted using Statistical Package for the Social Sciences (SPSS) (version 22.0; IBM Corp) and Stata (version 15.1; StataCorp) statistical software.

The intention-to-treat principle will be followed in the data analysis. However, an additional sensitivity analysis will be conducted to handle missing data during the analysis, where fully conditional multiple imputation with *n (% missing)* imputations and 1000 iterations using the Markov Chain Monte Carlo method will be used [67]. Model summary estimates will be calculated based on the Rubin rule [68].

#### Qualitative Data

All qualitative data are stored and analyzed using the NVivo (QSR International) computer software. Phenomenological analysis will be used to obtain an in-depth and comprehensive view of the data set from an insider’s perspective [69]. Unique or minority voices will be elicited to illuminate counterpoints to the stated views. Throughout the data analysis process, strategies to maximize research rigor and trustworthiness will be prioritized. The use of such a method of data analysis has been demonstrated in previous research involving grief therapy with bereaved parents [70].

### Ethical Considerations

This study has been approved by the institutional review board of Nanyang Technological University, Singapore (IRB-2018-07-009). Web-based, endorsed informed consent was obtained from all participants before study participation. Participants’ confidentiality, safety from unintended outcomes, and right to withdraw without any adverse consequences are safeguarded under the ethical provisions of Human Biomedical Research Act studies reviewed by the institutional review board of Nanyang Technological University, Singapore.

As such, there is minimal risk of engaging in a web-based narrative writing activity. The experienced NeW-I therapist will be available to participants to offer web-based support, in the event that some aspects of the intervention cause them distress or discomfort. If participants need further support, a referral system has been set up such that participants who are recruited via purposive sampling would be referred to their health and social care provider for follow-up assistance. Finally, any deviations from or changes to the study protocol, unexpected breaches in privacy, or major technical difficulties will be promptly reported to the institutional review board of Nanyang Technological University, Singapore, and further steps will be taken after seeking advice from the board.

## Results

Foundational research leading to this study received funding support from the Singapore Ministry of Education in 2017, and further funding support for pilot implementation of the intervention was obtained from the Temasek Foundation Innovates’ Singapore Millennium Foundation Grant in 2018. The study was approved by the institutional review board of Nanyang Technological University in 2018. The recruitment of participants started in January 2019 and is ongoing. Data collection is expected to be completed in September 2020.

## Discussion

### Research Synopsis and Implications

NeW-I is a first-of-its-kind, web-based, therapist-facilitated, strength-focused, and meaning-oriented intervention for parents of children with chronic life-threatening illnesses, thereby filling in a critical service gap in local pediatric palliative care. Through an open-label randomized control trial, the efficacy of NeW-I for improving such parents’ quality of life, spiritual well-being, hope, and social support and decreasing depressive symptoms, caregiver burden, and risk of complicated grief is investigated across 5 time points. It is expected that parents who successfully complete the structured NeW-I intervention protocol will experience enhanced quality of life, spiritual well-being, sense of hope, and perceived social support and decreased depressive symptoms, subjective caregiver burden, and risk of complicated grief as compared with parents who engage in a journaling activity unrelated to their child’s illness (control group). It is also expected that parents will find NeW-I to be an accessible and user-friendly service.

In addition to improving the psychosociospiritual well-being of parent caregivers, this study creates opportunities for (1) a longitudinal assessment of the mental states of 36 parents (ie, control group) to obtain a naturalistic trajectory of anticipatory grief, (2) an evaluation of the extent to which the intervention is successful in improving the mental well-being of 36 parents (ie, intervention group), and (3) an evaluation of the extent to which these potential positive effects are sustained over time. The findings will inform the development of a full-scale NeW-I protocol that will be disseminated via research papers and presentations. This will form the foundation for further empirical research to test the effectiveness, acceptability, and feasibility of NeW-I in Singapore and in other Asian communities around the world.

The web-based narrative writing model of NeW-I and the relative anonymity it offers to participants [43] supports the unique needs of Asian family caregivers who are uncomfortable with emotional expression even during times of loss and separation [47]. It is hoped that NeW-I is perceived by parents to be a safe platform for engaging in intimate dialogue regarding their child’s caregiving, thereby enhancing parents’ experience of their child’s illness trajectory, empowering them to harness available resources to provide the best possible care to their child, while simultaneously reducing psychosocial distress and caregiver burden. Evidence from a recent systematic review [71] suggests that the guided web-based intervention protocol of NeW-I is likely to be cost-effective. Additionally, the web-based intervention protocol is convenient to access, allows expression of disenfranchised emotions, and promotes meaning ascription to traumatic experiences. Finally, the current format of NeW-I is tailored for parents of children with chronic life-threatening illnesses. However, after a detailed examination of the structural and implementation strengths and challenges of NeW-I, its effectiveness in enhancing mental health as well as feasibility and accessibility, the web-based therapeutic protocol can be adapted to deliver psychotherapy to diverse populations including young adults who are diagnosed with a life-limiting condition, siblings of terminally ill young persons, and caregivers of patients with dementia, to name a few.

### Limitations and Conclusions

Presently, NeW-I can only be implemented with participants who speak, read, and write English. Singapore is a multicultural and multilinguistic nation [72], and future research should expand the delivery language of NeW-I and assess its acceptability and effectiveness among different linguistic and ethnic groups in Singapore and globally. Despite this limitation, NeW-I could enhance participants’ wellness by drawing attention away from their illness narrative and instead emphasize areas that research has demonstrated to be most meaningful at the end of life. Expected study outcomes can generate new knowledge to inform research and practice in pediatric palliative care and parental bereavement support locally and globally.

## Appendix

Multimedia Appendix 1 Screenshots of the narrative e-writing intervention app.

Multimedia Appendix 2 CONSORT‐EHEALTH checklist (V 1.6.1).

## Abbreviations

BGQ: brief grief questionnaire
BSFC-s: Burden Scale for Family Caregivers-Short
FACIT-Sp-12: Functional Assessment of Chronic Illness Therapy - Spiritual Well-Being scale
HHI: Herth hope index
ISS: inventory of social support
K-10: Kessler psychological distress scale
KQOL: Kemp quality of life scale
NeW-I: Narrative e-Writing Intervention
PHQ-9: Patient Health Questionnaire – 9
