# Correction: A Novel Narrative E-Writing Intervention for Parents of Children With Chronic Life-Threatening Illnesses: Protocol for a Pilot, Open-Label Randomized Controlled Trial

**DOI:** 10.2196/22286

**Published:** 2020-08-18

**Authors:** Andy Hau Yan Ho, Oindrila Dutta, Geraldine Tan-Ho, Toh Hsiang Benny Tan, Xinyi Casuarine Low, Sashikumar Ganapathy, Josip Car, Ringo Moon-Ho Ho, Chun Yan Miao

**Affiliations:** 1 Psychology Program School of Social Sciences Nanyang Technological University Singapore Singapore; 2 Lee Kong Chian School of Medicine Nanyang Technological University Singapore Singapore; 3 Palliative Care Centre for Excellence in Research and Education Singapore Singapore; 4 School of Computer Science and Engineering Nanyang Technological University Singapore Singapore; 5 Club Rainbow Singapore Singapore; 6 Centre for Population Health Sciences Lee Kong Chian School of Medicine Nanyang Technological University Singapore Singapore

In “A Novel Narrative E-Writing Intervention for Parents of Children With Chronic Life-Threatening Illnesses: Protocol for a Pilot, Open-Label Randomized Controlled Trial” (JMIR Res Protoc 2020;9(7):e17561) the authors noted several errors.

In the original paper, an incorrect telephone number was provided for the corresponding author. The telephone number has been corrected to 65 63168943.

One of the author names was incorrectly displayed as “Casuarine Xinyi Low”. It has now been corrected to:

Xinyi Casuarine Low

The correction will appear in the online version of the paper on the JMIR Publications website on August 18, 2020, together with the publication of this correction notice. Because this was made after submission to PubMed, PubMed Central, and other full-text repositories, the corrected article has also been resubmitted to those repositories.

