# Supplementary material for: A Novel Narrative E-Writing Intervention for Parents of Children With Chronic Life-Threatening Illnesses: Protocol for a Pilot, Open-Label Randomized Controlled Trial
Source: JMIR Res Protoc. 2020 Jul 5;9(7):e17561. doi: 10.2196/17561 (PMC7380996; doi:10.2196/17561)
Supplement: Multimedia Appendix 1 [file resprot_v9i7e17561_app1.pdf]

Multimedia Appendix 1. Screenshots of NeW-I app

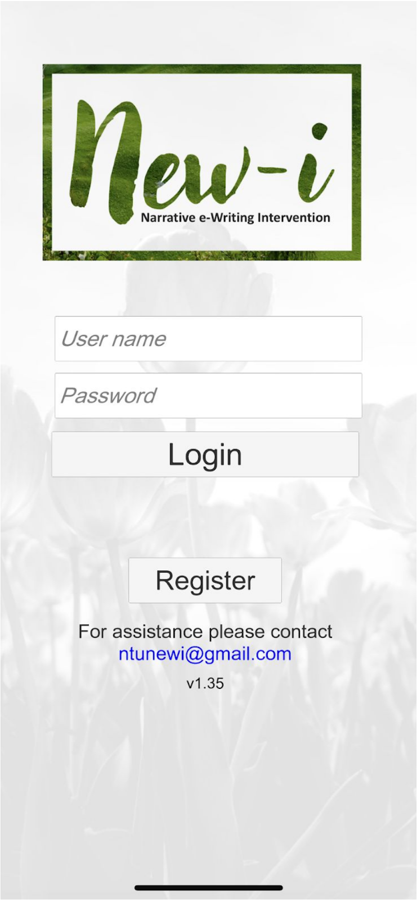

(1) Landing page of the app

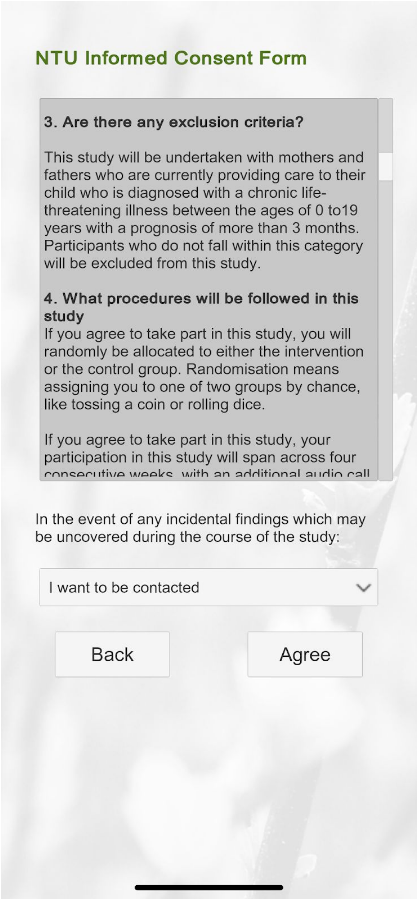

(2) Informed consent page

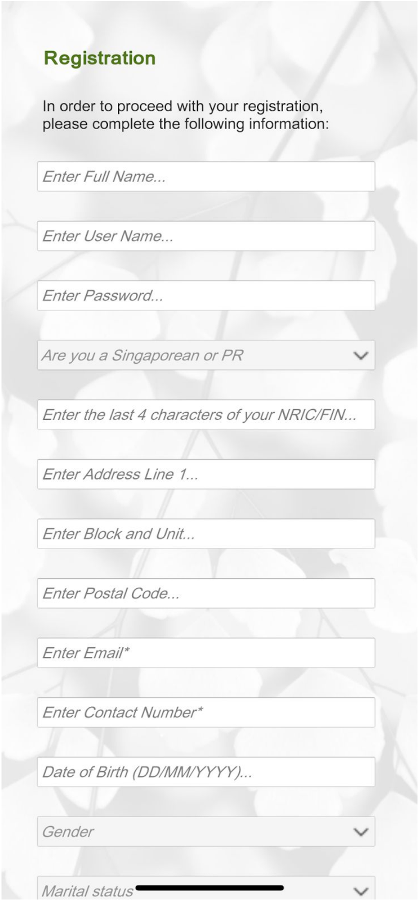

(3) Registration and demographic information page
