# Supplementary material for: A Novel Narrative E-Writing Intervention for Parents of Children With Chronic Life-Threatening Illnesses: Protocol for a Pilot, Open-Label Randomized Controlled Trial
Source: JMIR Res Protoc. 2020 Jul 5;9(7):e17561. doi: 10.2196/17561 (PMC7380996; doi:10.2196/17561)
Supplement: Multimedia Appendix 2 [file resprot_v9i7e17561_app2.pdf]

CONSORT-EHEALTH checklist (V.1.6.1)

Eysenbach, G. (2011). CONSORT-EHEALTH: improving and standardizing evaluation reports of Web-based and mobile health interventions. Journal of Medical Internet Research, 13(4), e126. https://doi.org/10.2196/jmir.1923

Item No.	CONSORT EHEALTH Checklist Item	Reported in manuscript / Relevant sections from manuscript
**TITLE**		
1a-i	Identify the mode of delivery in the title. Preferably use "web-based" and/or "mobile" and/or "electronic game" in the title. Avoid ambiguous terms like "online", "virtual", "interactive". Use "Internet-based" only if Intervention includes non-web-based Internet components (e.g., email),<br>use "computer-based" or "electronic" only if offline products are used. Use "virtual" only in the context of "virtual reality" (3-D worlds). Use "online"<br>only in the context of "online support groups". Complement or substitute product names with broader terms for the class of products (such as "mobile" or "smartphone" instead of "iphone"), especially if the application runs on different platforms.	Yes.<br><br>"Narrative E-Writing Intervention".<br><br>We prefer to use the term 'e' which indicates that the intervention can be delivered via both a phone app and web based platform.
1a-ii	Mention non-web-based components or important co-interventions<br>in the title, if any (e.g., "with telephone support").	NA since this is a methods and protocol manuscript.
1a-iii	Mention primary condition or target group in the title, if any (e.g., "for children with Type I Diabetes")	Yes.<br><br>"for Parents of Children with Chronic Life-Threatening Illnesses"
**ABSTRACT**		
1b-i	Mention key features/functionalities/components of the intervention	Yes.<br><br>"NeW-I, a strengths-and-meaning-focused and

	and comparator in the abstract. If possible, also mention theories and principles used for designing the site. Keep in mind the needs of systematic reviewers and indexers by including important synonyms.	therapist-facilitated mobile app and web-based counseling platform…"
1b-ii	Clarify the level of human involvement in the abstract, e.g., use phrases like "fully automated" vs. "therapist/nurse/care provider/physician-assisted" (mention number and expertise of providers involved, if any).	Yes.<br><br>"therapist-facilitated mobile app and web-based counseling platform…"
1b-iii	Open vs. closed, web-based (self-assessment) vs. face-to-face assessments in abstract: Mention how participants were recruited (online vs. offline), e.g., from an open access website or from a clinic or a closed online user group (closed user group trial), and clarify if this was a purely web-based trial, or there were face-to-face components (as part of the intervention or for assessment). Clearly say if outcomes were self assessed through questionnaires (as common in web-based trials). Note: In traditional offline trials, an open trial (open-label trial) is a type of clinical trial in which both the researchers and participants know which treatment is being administered. To avoid confusion, use "blinded" or "unblinded" to indicated the level of blinding instead of "open", as "open" in web-based trials usually refers to "open access" (i.e. participants can self-enrol)	Yes.<br><br>"NeW-I is implemented in Singapore as a pilot open-label randomized controlled trial comprising an intervention and control group. Both primary and secondary outcomes are self-reported by participants through questionnaires."
1b-iv	Results in abstract must contain use data: Report number of participants enrolled/assessed in each group, the use/uptake of the intervention (e.g., attrition/adherence metrics, use over time, number of logins etc.), in addition to primary/secondary	Yes.<br><br>"Funding support and IRB approval for this study has been secured. Data collection started in January 2019 and is ongoing."

	outcomes. (Note: Only report in the abstract what the main paper is reporting. If this information is missing from the main body of text, consider adding it)	
1b-v	Conclusions/Discussions in abstract for negative trials: Discuss the primary outcome - if the trial is negative (primary outcome not changed), and the intervention was not used, discuss whether negative results are attributable to lack of uptake and discuss reasons.	We have included a discussion section, but negative results have not been found as this paper is a methods and protocol manuscript that is not reporting results.
**INTRODUCTION**		
2a-i	Describe the problem and the type of system/solution that is object of the study: intended as stand-alone intervention vs. incorporated in broader health care program? [1] Intended for a particular patient population? [1] Goals of the intervention, e.g., being more cost-effective to other interventions [1], replace or complement other solutions?	Yes.<br><br>"there is a need to develop a pre-loss intervention to augment pediatric palliative care and parental bereavement support service - one that empowers parents to reflect on their caregiving experiences, explore and identify resources that could help them better cope with the challenges of caregiving, and support their child to live a meaningful life despite a chronic life-threatening illness."<br><br>"Globally, pediatric palliative care interventions predominantly emphasize the stages of grief and psychological tasks that grieving parents must accomplish after their child's death, and in Singapore, there is no known empirically-tested intervention to provide psycho-emotional support and psychoeducational resources to parents of children with chronic life-threatening illness."<br><br>"NeW-I is a novel internet-based, therapist-facilitated, strength-focused, and meaning-oriented intervention designed to provide direct service to

		parents facing their child's chronic life-threatening illness."
2a-ii	Scientific background, rationale: What is known about the (type of) system that is the object of the study (be sure to discuss the use of similar systems for other conditions/diagnoses, if appropriate), motivation for the study, i.e., what are the reasons for and what is the context for this specific study, from which stakeholder viewpoint is the study performed, potential impact of findings [2]. Briefly justify the choice of the comparator.	Yes.<br><br>"The development and evaluation of NeW-I is guided by the Medical Research Council Framework for the Development and Evaluation of Complex Interventions which is widely recognized in the design and evaluation of complex interventions to improve health outcomes. NeW-I is also inspired by the meaning-reconstruction model, the narrative approach to anticipatory grief, dignity therapy, family dignity intervention for holistic end-of-life care, and the findings of a recent investigation on Asian parental bereavement experience of child loss by our research team."
2b	Specific objectives or hypotheses	Yes.<br><br>"The overarching goal of the novel intervention model 'NeW-I' is to provide a new eye or a new perspective for caregiver-parents to look at their own experiences and narrative, thereby resulting in the creation of a New I, a restructured understanding of the self in the context of their experiences. This overarching goal can be deconstructed into four key objectives, which are: (1) To develop a pilot study protocol for a culture-specific and meaning-oriented Narrative e-Writing Intervention (NeW-I) for anticipatory grief and bereavement support for Asian parents facing their child's chronic life-threatening illness and impending death; (2) To evaluate the efficacy of NeW-I in enhancing quality of life, spiritual well-being, hope and perceived social support, and decreasing depressive symptoms, caregiver burden and risk of complicated grief among participants; (3) To assess the

		acceptability and feasibility of implementing NeW-I among Asian parents of children with chronic life-threatening illness in Singapore; and (4) To develop a standardized protocol for further empirical research to test the effectiveness, acceptability and feasibility of NeW-I in Singapore and in other Asian communities around the world.”
**METHODS**		
3a	Description of trial design (such as parallel, factorial) including allocation ratio	Yes.<br><br>“The present study adopts an open-label randomized controlled trial design comprising two groups: (1) an intervention group (structured NeW-I protocol) and (2) a control group (journaling activity unrelated to their child’s illness). It is hypothesized that intervention participants who successfully complete NeW-I will experience enhanced quality of life, spiritual well-being, sense of hope and perceived social support, and decreased depressive symptoms, subjective caregiver burden and risk of complicated grief as compared to control participants. It is also hypothesized that NeW-I is deemed an accessible and user-friendly service by participants.”
3b-i	Bug fixes, Downtimes, Content Changes: ehealth systems are often dynamic systems. A description of changes to methods therefore also includes important changes made on the intervention or comparator during the trial (e.g., major bug fixes or changes in the functionality or content) (5-iii) and other “unexpected events” that may have influenced study design	We do not have major bug fixes, downtimes, content changes or unexpected events to the NeW-I app to report

4a-i	Computer / Internet literacy is often an implicit “de facto” eligibility criterion - this should be explicitly clarified	Yes.<br><br>“Singapore is a leading nation in digital readiness, smartphone utilization for communication is ingrained into the everyday life of its people, and mobile-based intervention services for improving well-being have been welcomed by the Singapore community. Hence, it is reasonable to propose that internet-based solutions could be vital in enhancing pediatric palliative care and parental bereavement support services.”
4a-ii	Open vs. closed, web-based vs. face-to-face assessments: Mention how participants were recruited (online vs. offline), e.g., from an open access website or from a clinic, and clarify if this was a purely web-based trial, or there were face-to-face components (as part of the intervention or for assessment), i.e., to what degree the study team got to know the participant. In online-only trials, clarify if participants were quasianonymous and whether having multiple identities was possible or whether technical or logistical measures (e.g., cookies, email confirmation, phone calls) were used to detect/prevent these.	Yes.<br><br>“Potential participants are identified and contacted by the collaborating organization to introduce the study to their beneficiaries. If verbal consent is obtained from potential participants, their contact details are passed to the research team at Nanyang Technological University, who subsequently establish telephone contact, explain study procedures and introduce the NeW-I online platform.<br>Open recruitment is also carried out, so that all parents of children with chronic life-threatening illness have equal opportunity to participate in a potentially beneficial study.<br><br>“Participants are requested to provide their unique contact details and national identification number to ensure that only one user account is created by each individual.”
4a-iii	Information given during recruitment. Specify how participants were briefed for recruitment and in the informed consent procedures (e.g., publish the informed consent documentation as appendix, see also item X26), as this information may have an effect on	Yes.<br><br>“When interested participants contact the research team, study procedures are explained, the NeW-I platform is introduced, and registration information is provided.”

	user self-selection, user expectation and may also bias results	"When participants initially log on to the app or website, they are directed to a study participation and informed consent page that provides details about study procedures, institutional affiliations of the research team, rights of research participants and protection of confidentiality."
4b-i	Clearly report if outcomes were (self-)assessed through online questionnaires (as common in web-based trials) or otherwise.	Yes.<br><br>"Via the NeW-I platform, both intervention and control group participants fill out a socio-demographic form at baseline and are then assessed on a battery of self-reported standardized and validated measures across 5 time-points."
4b-ii	Report how institutional affiliations are displayed to potential participants [on ehealth media], as affiliations with prestigious hospitals or universities may affect volunteer rates, use, and reactions with regards to an intervention" [1].(Not a required item – describe only if this may bias results)	Yes.<br><br>"When participants initially log on to the app or website, they are directed to a study participation and informed consent page that provides details about institutional affiliations of the research team…"
5-i	Mention names, credentials, affiliations of the developers, sponsors, and owners [6] (if authors/ evaluators are owners or developers of the software, this needs to be declared in a "Conflict of interest" section or mentioned elsewhere in the manuscript).	Yes.<br><br>This information is mentioned in the title page and under Declarations.
5-ii	Describe the history/development process of the application and previous formative evaluations (e.g., focus groups, usability testing), as these will have an impact on adoption/use rates and help with interpreting results.	Yes.<br><br>Information about foundational studies that empirically informed the development of this intervention is mentioned under Parental Bereavement Trajectories of Child Loss.

		Since this is the protocol of a pilot trial, there have been no prior evaluations of the intervention described here.
5-iii	Revisions and updating. Clearly mention the date and/or version number of the application/intervention (and comparator, if applicable) evaluated, or describe whether the intervention underwent major changes during the evaluation process, or whether the development and/or content was "frozen" during the trial. Describe dynamic components such as news feeds or changing content which may have an impact on the replicability of the intervention (for unexpected events see item 3b).	We do not have major revisions, updating, or content changes to the intervention design or to the NeW-I app to report.
5-iv	Provide information on quality assurance methods to ensure accuracy and quality of information provided [1], if applicable.	Yes.<br><br>"All feedback provided to participants is vetted by at least two members of the research team for data monitoring, quality and safety assurance."
5-v	Ensure replicability by publishing the source code, and/or providing screenshots/screen-capture video, and/or providing flowcharts of the algorithms used. Replicability (i.e., other researchers should in principle be able to replicate the study) is a hallmark of scientific reporting.	NA.<br><br>We believe that the novelty and essence of NeW-I lies in the content and empirical foundation of the intervention model. As such, the app is simple and straightforward and could be developed by any individual who is experienced in app development. Hence, we do not feel that this information would be valuable to the readers of this article.
5-vi	Digital preservation: Provide the URL of the application, but as the intervention is likely to change or disappear over the course of the years; also make sure the intervention is archived (Internet Archive, webcitation.org, and/or publishing the source code or screenshots/videos alongside the article). As pages behind login screens cannot be archived,	NA.<br><br>NeW-I is still a pilot intervention at the present time. We believe it is appropriate to wait until a full-scale NeW-I app is developed and implemented, and then provide the URL for public access.

	consider creating demo pages which are accessible without login.	
5-vii	Access: Describe how participants accessed the application, in what setting/context, if they had to pay (or were paid) or not, whether they had to be a member of a specific group. If known, describe how participants obtained "access to the platform and Internet" [1]. To ensure access for editors/reviewers/readers, consider to provide a "backdoor" login account or demo mode for reviewers/readers to explore the application (also important for archiving purposes, see vi).	Yes.<br><br>"Participants can download the app free-of-cost from Apple App Store and Google Play Store by keying in the relevant keywords or scanning the QR code provided on NeW-I study advertisement posters."
5-viii	Describe mode of delivery, features/functionalities/components of the intervention and comparator, and the theoretical framework [6] used to design them (instructional strategy [1], behaviour change techniques, persuasive features, etc., see e.g., [7, 8] for terminology). This includes an in-depth description of the content (including where it is coming from and who developed it) [1], "whether [and how] it is tailored to individual circumstances and allows users to track their progress and receive feedback" [6]. This also includes a description of communication delivery channels and – if computer-mediated communication is a component – whether communication was synchronous or asynchronous [6]. It also includes information on presentation strategies [1], including page design principles, average amount of text on pages, presence of hyperlinks to other resources etc. [1]	Yes.<br><br>In-depth description of the content and it's theoretical framework is mentioned in Table 1.<br>Communication between the NeW-I therapist and the participant is asynchronous and computer-mediated, as shown in Figure 2.
5-ix	Describe use parameters (e.g., intended "doses" and optimal timing for use) [1]. Clarify what instructions or recommendations were given to the user, e.g., regarding timing,	Yes.<br><br>"Both intervention and control group participants follow the procedures described in Figure 2. There are 4

	frequency, heaviness of use [1], if any, or was the intervention used ad libitum.	weekly sessions of writing. A template is provided to ensure that participants' writings tie in with the session objectives. To improve participants' adherence to the study protocol, they receive an automated notification on their phone app and email each time a fresh writing session becomes available to them."
5-x	Clarify the level of human involvement (care providers or health professionals, also technical assistance) in the e-intervention or as co-intervention. Detail number and expertise of professionals involved, if any, as well as "type of assistance offered, the timing and frequency of the support, how it is initiated, and the medium by which the assistance is delivered" [6]. It may be necessary to distinguish between the level of human involvement required for the trial, and the level of human involvement required for a routine application outside of a RCT setting (discuss under item 21 – generalizability)	Yes.<br><br>"NeW-I is delivered by trained therapists in the research team who are experts in death education and grief counselling and have the clinical competence to work with family caregivers in pediatric palliative settings."
5-xi	Report any prompts/reminders used: Clarify if there were prompts (letters, emails, phone calls, SMS) to use the application, what triggered them, frequency, etc. [1]. It may be necessary to distinguish between the level of prompts/reminders required for the trial, and the level of prompts/reminders for a routine application outside of a RCT setting (discuss under item 21 – generalizability).	Yes.<br><br>To improve participants' adherence to the study protocol, they receive an automated notification on their phone app and email each time a fresh writing session becomes available to them.
5-xii	Describe any co-interventions (incl. training/support): Clearly state any "interventions that are provided in addition to the targeted eHealth intervention" [1], as ehealth intervention may not be designed as	Although potentially important in other investigations, no co-interventions took place during this study.

	standalone intervention. This includes training sessions and support [1]. It may be necessary to distinguish between the level of training required for the<br>trial, and the level of training for a routine application outside of a RCT setting (discuss under item 21 – generalizability.	
6a-i	If outcomes were obtained through online questionnaires, describe if they were validated for online use [6] and apply CHERRIES items to describe how the questionnaires were designed/deployed [9].	Yes, outcomes were obtained through online questionnaires. These questionnaires involve a battery of self-reported standardized and validated measures that are commonly used in conventional physical settings, but are not known to have been validated for online usage.<br><br>See the CHERRIES checklist included as a supplementary file for description of how the questionnaires were designed/deployed.
6a-ii	Describe whether and how "use" (including intensity of use/dosage) was defined/measured/monitored (logins, log file analysis, etc.). Use/adoption metrics are important process outcomes that should be reported in any ehealth trial.	This may be relevant in other studies, but for the NeW-I trial, participants' use of the app is limited to once-a-week writing sessions for 4 consecutive weeks, reading of the therapist's responses, and completing the 5 assessments. Hence, participants' usage of the app was not monitored.
6a-iii	Describe whether, how, and when qualitative feedback was obtained from participants (e.g., through emails, feedback forms, interviews, focus groups).	Yes.<br><br>"To evaluate the acceptability and effectiveness of NeW-I, all intervention participants are invited to participate in a semi-structured interview at the completion of all intervention components at T2, which explores the impact of the intervention, aspects of the intervention found to be helpful, aspects of the intervention found to be unhelpful and how they could be improved, challenges encountered in completing the intervention, and scope for enhancing intervention usability."

6b	Any changes to trial outcomes after the trial commenced, with reasons	
7a-i	Describe whether and how expected attrition was taken into account when calculating the sample size	Yes.<br><br>"The trial aims to enrol 36 participants in each group (N=72), so that allowing for 30% attrition at follow-up, the sample size is adequate to detect a small effect size of 0.2 in the primary outcome measure with 90% power and P<.05 (two-sided)."
7b	When applicable, explanation of any interim analyses and stopping guidelines	Interim analyses are not applicable since this is a methods and protocol manuscript.<br><br>As such, there are no stopping guidelines since there is minimal risk for engaging in an online narrative writing activity. However, measures have been put in place to protect participants' safety, which are as follows:<br>1) The experienced NeW-I therapist will be available to participants to offer online support, in the event that some aspects of the intervention cause them distress or discomfort.<br>2) If participants need further support, a referral system has been set up such that participants who are recruited via purposive sampling would be referred to their health-and-social care provider for follow-up assistance.<br>3) Finally, any deviations from or changes to the study protocol, unexpected breaches in privacy or major technical difficulties will be promptly reported to the Institutional Review Board of Nanyang Technological University Singapore and further steps will be taken after seeking advice from the Board."
8a	Method used to generate the random allocation sequence<br>NPT: When applicable, how care providers were allocated to each	Yes.<br><br>"random allocation of participants to either the intervention or the control

	trial group	group which is done via the NeW-I platform by using computer-generated random numbers.”
8b	Type of randomisation; details of any restriction (such as blocking and block size)	NA.
9	Mechanism used to implement the random allocation sequence (such as sequentially numbered containers), describing any steps taken to conceal the sequence until interventions were assigned.	NA.
10	Who generated the random allocation sequence, who enrolled participants, and who assigned participants to interventions	Yes.<br><br>The random allocation sequence was generated by the NeW-I app, participants enrolled on the NeW-I app voluntarily by completing a registration form and screening survey. Participants were assigned to either intervention or control group randomly by the NeW-I app.
11a-i	Specify who was blinded, and who wasn’t. Usually, in web-based trials it is not possible to blind the participants [1, 3] (this should be clearly acknowledged), but it may be possible to blind outcome assessors, those<br>doing data analysis or those administering co-interventions (if any).	NA since this is an open-label trial.
11a-ii	Informed consent procedures (4a-ii) can create biases and certain expectations - discuss e.g., whether participants knew which intervention was the “intervention of interest” and which one was the<br>“comparator”.	Yes.<br><br>Since this is an open-label study, participants are informed whether they have been allocated to the intervention or control group via the NeW-I app.
12a-i	Imputation techniques to deal with attrition / missing values: Not all participants will use the intervention/comparator as intended	Yes.<br><br>This trial allows for an attrition rate of 30% at follow-up (a larger estimate due

	and attrition is typically high in ehealth trials. Specify how participants who did not use the application or dropped out from the trial were treated in the statistical analysis (a complete case analysis is strongly discouraged, and simple imputation techniques such as LOCF may also be problematic [4]).	to end-of-life context). Hence, the target sample size has been inflated by a factor of 1 / (1-0.3) = 1.43.<br><br>The data analysis plan involves a sensitivity analysis to handle missing data, where fully conditional multiple imputation with 'n (% missing)' imputations and 1000 iterations using Markov Chain Monte Carlo method will be used.
12b	Methods for additional analyses, such as subgroup analyses and adjusted analyses	NA.
x26-i	Comment on ethics committee approval.	Yes.<br><br>"This study has been approved by the Institutional Review Board of Nanyang Technological University Singapore (IRB-2018-07-009)."
x26-ii	Outline informed consent procedures e.g., if consent was obtained offline or online (how? Checkbox, etc.?), and what information was provided (see 4a-ii). See [6] for some items to be included in informed consent documents	Yes.<br><br>"When participants initially log on to the app or website, they are directed to a study participation and informed consent page that provides details about study procedures, institutional affiliations of the research team, rights of research participants and protection of confidentiality." Only after participants endorse this online informed consent form, they can proceed with the different study components.
x26-iii	Safety and security procedures, incl. privacy considerations, and "any steps taken to reduce the likelihood or detection of harm (e.g., education and training, availability of a hotline)" [1].	Yes.<br><br>Information collected for this study will be kept confidential and stored for a minimum of 10 years in a secure environment with restricted access within NTU. In the event of any publication regarding this study, only aggregated research data without identifiable personal details will be

		used, and participants' identity will remain confidential.
**RESULTS**		
13a	For each group, the numbers of participants who were randomly assigned, received intended treatment, and were analysed for the primary outcome NPT: The number of care providers or centers performing the intervention in each group and the number of patients treated by each care provider in each center	NA since this is a methods and protocol manuscript.
13b-i	Strongly recommended: An attrition diagram (e.g., proportion of participants still logging in or using the intervention/comparator in each group plotted over time, similar to a survival curve) [5] or other figures or tables demonstrating usage/dose/engagement.	NA since this is a methods and protocol manuscript.
14a-i	Indicate if critical "secular events" [1] fell into the study period, e.g., significant changes in Internet resources available or "changes in computer hardware or Internet delivery resources" [1].	NA since this is a methods and protocol manuscript.
14b	Why the trial ended or was stopped [early].	NA since this is a methods and protocol manuscript.
15-i	In ehealth trials it is particularly important to report demographics associated with digital divide issues, such as age, education, gender, social-economic status, computer/Internet/ehealth literacy of the participants, if known.	NA since this is a methods and protocol manuscript.
16-i	Report multiple "denominators" and provide definitions: Report N's (and effect sizes) "across a range of study participation [and use] thresholds" [1], e.g., N exposed, N consented, N used more than x	NA since this is a methods and protocol manuscript.

	times, N used more than y weeks, N participants "used" the intervention/comparator at specific predefined time points of interest (in absolute and relative numbers per group). Always clearly define "use" of the intervention.	
16-ii	Primary analysis should be intent-to-treat; secondary analyses could include comparing only "users", with the appropriate caveats that this is no longer a randomized sample (see 18-i).	Yes.<br><br>"The intention-to-treat principle will be followed in data analysis."
17a-i	In addition to primary/secondary (clinical) outcomes, the presentation of process outcomes such as metrics of use and intensity of use (dose, exposure) and their operational definitions is critical. This does not only refer to metrics of attrition (13-b) (often a binary variable), but also to more continuous exposure metrics such as "average session length". These must be accompanied by a technical description how a Highly Recommended CONSORT EHEALTH 2011 checklist Page 11 metric like a "session" is defined (e.g., timeout after idle time) [1] (report under item 6a).	NA since all participants engage in 4 sessions of consecutive weekly writing and all sessions must be completed within 30 mins.
17b	For binary outcomes, presentation of both absolute and relative effect sizes is recommended	NA since this is a methods and protocol manuscript.
18-i	Subgroup analysis of comparing only users is not uncommon in ehealth trials, but if done it must be stressed that this is a self-selected sample and no longer an unbiased sample from a randomized trial (see 16-iii).	NA since this is a methods and protocol manuscript.
19-i	Include privacy breaches, technical problems. This does not only include physical "harm" to participants, but also incidents such as	Yes.<br><br>"any deviations from or changes to the study protocol, unexpected breaches in

	perceived or real privacy breaches [1], technical problems, and other unexpected/unintended incidents. “Unintended effects” also includes unintended positive effects [2].	privacy or major technical difficulties will be promptly reported to the Institutional Review Board of Nanyang Technological University Singapore and further steps will be taken after seeking advice from the Board.”
19-ii	Include qualitative feedback from participants or observations from staff/researchers, if available, on strengths and shortcomings of the application, especially if they point to unintended/unexpected effects or uses. This includes (if available) reasons for why people did or did not use the application as intended by the developers.	Yes.<br><br>“To evaluate the acceptability and effectiveness of NeW-I, all intervention participants are invited to participate in a semi-structured interview at the completion of all intervention components, which explores the impact of the intervention, aspects of the intervention found to be helpful, aspects of the intervention found to be unhelpful and how they could be improved, challenges encountered in completing the intervention, and scope for enhancing intervention usability.”
22-i	Restate study questions and summarize the answers suggested by the data [2], starting with primary outcomes and process outcomes (use).	Yes.<br><br>We have included a brief discussion section where we summarize the research framework and expected outcomes, but we do not summarize the data since this is a methods and protocol manuscript.
22-ii	Highlight unanswered new questions, suggest future research [2]	NA since this is a methods and protocol manuscript.
	**DISCUSSION**	
20-i	Typical limitations in ehealth trials: Participants in ehealth trials are rarely blinded. Ehealth trials often look at a multiplicity of outcomes, increasing risk for a Type I error. Discuss biases due to non-use of the intervention/usability issues, biases through informed consent procedures, unexpected events.	Yes.<br><br>We have discussed foreseeable limitations of this study at the end of the discussion section.
21-i	Generalizability to other populations: In particular, discuss	Yes.

	generalizability to a general Internet population, outside of a RCT setting, and general patient population, including applicability of the study results for other organizations [2].	“the current format of NeW-I is tailored for parents facing their child’s chronic life-threatening illness, however, after a detailed examination of the structural and implementation strengths and challenges of NeW-I, it’s effectiveness in enhancing mental health as well as feasibility and accessibility, the online therapeutic protocol can be adapted to deliver psychotherapy to diverse populations including young adults who are diagnosed with a life-limiting condition, siblings of terminally ill young persons and caregivers of patients with dementia, to name a few.”
21-ii	Discuss if there were elements in the RCT that would be different in a routine application setting (e.g., prompts/reminders, more human involvement, training sessions or other co-interventions) and what impact the omission of these elements could have on use, adoption, or outcomes if the intervention is applied outside of a RCT setting.	NA since this is a methods and protocol manuscript.
**OTHER INFORMATION**		
23	Registration number and name of trial registry	Yes.<br><br>“Trial registration: Registration: Clinicaltrials.gov NCT03684382; Verified: 25 September 2018; https://clinicaltrials.gov/ct2/show/NCT03684382”
24	Where the full trial protocol can be accessed, if available	This manuscript is meant to be the full trial protocol.
25	Sources of funding and other support (such as supply of drugs), role of funders	Yes.<br><br>“This work was supported by the Singapore Ministry of Education Academic Research Tier 1 Fund (2017-T1-001-034) and the Temasek Foundation Innovates’ Singapore Millennium Foundation Grant (M4062472.SS0). The funders played

		no role in the study design, collection, analysis or interpretation of data, or preparation of the manuscript.”
x27-i	In addition to the usual declaration of interests (financial or otherwise), also state the “relation of the study team towards the system being evaluated” [2], i.e., state if the authors/evaluators are distinct from or identical with the developers/sponsors of the intervention.	All parties involved were university affiliated researchers or community-based collaborators. All pa
